# Health Literacy, Socio-Economic Determinants, and Healthy Behaviours: Results from a Large Representative Sample of Tuscany Region, Italy

**DOI:** 10.3390/ijerph182312432

**Published:** 2021-11-26

**Authors:** Patrizio Zanobini, Chiara Lorini, Vieri Lastrucci, Valentina Minardi, Valentina Possenti, Maria Masocco, Giorgio Garofalo, Giovanna Mereu, Guglielmo Bonaccorsi

**Affiliations:** 1 Department of Health Sciences, University of Florence, 50134 Florence, Italy; chiara.lorini@unifi.it (C.L.); guglielmo.bonaccorsi@unifi.it (G.B.); 2 Epidemiology Unit, Meyer Children’s University Hospital, 50139 Florence, Italy; vieri.lastrucci@unifi.it; 3 Centro Nazionale per la Prevenzione delle Malattie e la Promozione della Salute, Istituto Superiore di Sanità, 00161 Rome, Italy; valentina.minardi@iss.it (V.M.); valentina.possenti@iss.it (V.P.); maria.masocco@iss.it (M.M.); 4 Department of Prevention, Azienda Unità Sanitaria Locale (AUSL) Toscana Centro, 50122 Florence, Italy; giorgio.garofalo@uslcentro.toscana.it (G.G.); giovanna.mereu@uslcentro.toscana.it (G.M.)

**Keywords:** health literacy, nutrition, physical activity, health behaviours

## Abstract

Background: Health Literacy (HL) is one of the main determinants of health and is crucial for the prevention of noncommunicable diseases, by influencing key health-related behaviours. The aim of the present study was to assess the role of HL and sociodemographic factors in predicting the adoption of two healthy behaviours—physical activity and fruits and vegetables consumption. Methods: This study was conducted on the Tuscan population subsample of the Italian Behavioral Risk Factor Surveillance System in 2017–2018. HL was assessed using the Italian version of the six-item European Health Literacy Survey Questionnaire (HLS-EU-Q6). Results: About 40% of the 7157 interviewees reported an inadequate or problematic HL level. Female sex, poor financial status, foreign nationality, and low education were associated with a problematic HL level, while an inadequate HL level was associated with being 50–69 years old, low education level, foreign nationality, poor financial status and unemployment or inactive status. Inadequate HL level was a strong predictor of both eating less than three portions of fruits/vegetables per day and not engaging in sufficient PA during leisure times. Conclusions: Our findings showed that an inadequate level of HL could negatively affect physical activity and diet, independently from the other sociodemographic conditions, confirming the role of HL as a relevant social determinant of health.

## 1. Introduction

Health literacy (HL) has been defined as “the combination of personal competencies and situational resources needed for people to access, understand, appraise and use information and services to make decisions about health. It includes the capacity to communicate, assert and act upon these decisions” [1]. 

In recent years, the number of published studies on HL has steadily increased and it is now considered as one of the main determinants of health and healthcare service use [2]. At present, low levels of HL are quite common: for instance, the results of the first European Health Literacy Survey (HLS-EU), conducted in 2011 in eight European Countries, showed that about 47% of respondents reported limited HL skills [3]. 

As recently described by the World Health Organization, HL is crucial for the prevention of noncommunicable diseases (NCDs) [4], by influencing key health-related behaviours such as physical activity (PA), fruit and vegetables consumption, tobacco use, and alcohol consumption [5,6]. According to the conceptual framework of Sørensen et al. [7], which encompasses the public health perspective, health behaviours are identified as consequences of HL. However, while most studies describe a linear relationship between level of HL and health behaviours [8,9,10], others find a limited association with only certain behaviours [11,12,13]. Moreover, the weight of HL in predicting health behaviours and risk factors differs according to the target populations included, the HL measurement tools used, and the heterogeneity in outcomes or study designs [5,6,14,15], thus reducing its usefulness for policymaking [16].

Since 2008, the surveillance system PASSI (Progressi delle Aziende Sanitarie per la Salute in Italia) has been collecting data on modifiable risk factors among the adult population living in Italy. Involving the Local Health Units (LHUs) under the supervision and coordination of the Italian National Institute of Health (namely, Istituto Superiore di Sanità; ISS), PASSI implements the model known as “behavioural risk factor surveillance” (BRFS) developed by the US Centers for Disease Prevention and Control. This approach is characterised by near-continuous data collection, while supporting health promotion and public health decision making by providing relevant information on behavioural risk factors [17]. Data are collected using a standardised questionnaire, whose core comprises more than one hundred questions grouped in 12 modules that investigate the lifestyles of adults (18–69 years) and the adherence to preventive measures and programmes. Over the years, the introduction of additional modules was proposed by regions or other institutions such as ministries or universities to answer specific informative needs on public health issues. Given the increased relevance assumed by HL, in 2017, an additional module to measure HL has been added to the core of the questionnaire of PASSI administered to the Tuscan population, as already experimented in other countries where similar surveillance systems or health surveys are ongoing [18,19]. The chosen assessment tool was the HLS-EU-Q6, a self-assessment questionary previously validated in Italy [20]. According to Pelikan et al. [21], it is an economic measure of HL in the general population, particularly suitable for large national surveys where comprehensive measures of HL, such as the HLS_EU-Q47, are too extensive and time consuming to be included.

In Italy, in fact, research on HL, its antecedents (i.e., factors affecting HL) and consequences (i.e., factors that are affected by HL) by using large, representative, population-based samples is still scarce [22], although understanding how HL impacts health behaviours would make it possible to focus interventions to mitigate the negative effects of low HL. Moreover, measuring HL at the population level can be considered as a prerequisite to improve organizational capacity for delivering HL services [23].

The aim of the present study was to evaluate the levels and the associations of HL in a representative sample of an adult general population living in the Tuscany Region, Italy. In particular, the study assessed the role of HL and sociodemographic factors in predicting the adoption of two healthy behaviours—physical activity and fruits and vegetables consumption. Furthermore, several possible socioeconomic determinants of HL were analysed.

## 2. Materials and Methods

### 2.1. Study Population, Sampling Criteria and Data Collection

This study is conducted on the Tuscan population subsample of the PASSI surveillance system. In each LHU participating in PASSI, a random sample is drawn monthly from the enrolment list of residents aged 18 to 69 years, stratified by sex and age proportionally to the size of the respective stratum in the general population. The exclusion criteria are: the unavailability of a phone number, the inability to speak and understand Italian, or to hold the interview (e.g., severe cognitive impairment, and severe disabilities), and hospitalization or institutionalization during the survey period.

Once the sample is extracted, a letter which explains the purpose of the surveillance system is sent to the selected persons. Telephone numbers are obtained from the enrolment list, telephone directories, the general practitioners (GPs), or the respondents themselves who, after receiving the letter, call for an appointment. At least six attempts are made to call on different days of the week, including weekends, and at different times of the day; after that, if a person cannot be reached, a substitute of the same sex and age group is randomly extracted.

Data are collected by trained personnel from the public health departments of each LHU using the computerised assisted telephone interview technique. Informed consent is obtained from all the interviewees. The data are thus anonymised and electronically recorded in a national database, and the interviews gathered during each calendar year are collected in an annual dataset, in order to obtain regional and national representative estimates. 

Further methodological details about PASSI have been described elsewhere [24,25,26].

The present study considered data referring to 7157 persons, which is the regional representative sample of Tuscany region residents in 2017–2018.

### 2.2. Measures

Sociodemographic characteristics included in this analysis were: sex, age (grouped in 18–34, 35–49, or 50–69 age groups), education level (dichotomized as: “low” for intermediate school diploma or lower; “high” for high school diploma or higher). Furthermore, economic status was assessed by asking if the respondents experience difficulties in making ends meet with the financial resources available (“With the financial resources at your disposal - from your own or family income - how do you get to the end of the month?” Four possible answers are provided and then grouped into two analytical categories: “very easily”, “quite easily” (coded together as “good”), “with some difficulties”, or “with many difficulties” (coded as “poor”). For occupational status, three categories were identified: employed, inactive and unemployed. Lastly, nationality (Italian or foreign) was considered.

HL level was assessed using the Italian version of the 6-item European Health Literacy Survey Questionnaire (HLS-EU-Q6) [20,27], which is the short-short form of the 47-item tool (HLS-EU-Q47). It is a self-reported tool with Likert-type responses (“very easy”, “fairly easy”, “fairly difficult”, “very difficult”) and a final score that can be used to measure HL in general populations. For each item, the following scores were considered: “very easy” = 4; “fairly easy” = 3; “fairly difficult” = 2; “very difficult” = 1. “Don’t know” or refusal were recorded as missing. The final scale score was the mean value and varied between 1 and 4. Only respondents who answered at least five items were considered. According to the final score (x), three possible levels of HL have been defined: inadequate HL (1 ≤ x ≤ 2); problematic HL (2 < x < 3); sufficient HL (3 ≤ x ≤ 4) [20,21]. 

Health behaviours considered included PA and fruits/vegetables consumption. 

Regarding PA, the respondents were asked if they adhere to the WHO guidelines on leisure-time PA (at least 150 min per week of physical activity, moderate or intense) [28]. Minutes of intense activity are worth twice as much as moderate activity and PA sessions of less than 10 min are equal to 0 min. Intense physical activity is defined as that which in amount, duration, and intensity cause a large increase in breathing and heart rate or profuse sweating, such as running, brisk cycling, aerobic exercise, or competitive sports. Moderate physical activity is defined as that which in amount, duration and intensity involve a slight increase in breathing and heart rate or some sweating, such as walking at a brisk pace, bicycling, gentle exercise, dancing, gardening or housework such as washing windows or floors.

Regarding diet, the respondent was asked how many portions of fruits or vegetables were routinely consumed per day. A portion of fruit or vegetables is defined as an amount of raw fruit or vegetables that can fit in the palm of a hand or half a plate of cooked vegetables. These portions roughly correspond to 80 g of fruit or vegetables.

In this study, diet was treated as a dichotomous variable depending on whether the respondent consumed at least three portions of fruits and/or vegetables per day.

### 2.3. Statistical Analysis

Descriptive data were presented as percentage or as mean and standard deviation. Complex survey design analyses using the Taylor series method for variance estimation were conducted. Percentage estimates were weighted, assigning each record a probability weight equal to the inverse of the sampling fraction in each LHU stratum [24]. Bivariate associations between HL level and sociodemographic factors were assessed using the Chi-squared test. To date, the exact nature of the relationship linking the level of HL to sociodemographic factors is not well known. Therefore, instead of an ordinal logistic model, which requires a proportional odds assumption, a multivariate multinomial logistic regression model was adopted to assess the association between sociodemographic factors (independent variables) and HL level (dependent variable, coded as inadequate, problematic or sufficient, considering sufficient as the reference category). The regression coefficients are expressed as relative risk ratio (RRR). Multivariate logistic regression models were used to evaluate the relationship of both PA and fruits/vegetables consumption with sociodemographic factors and HL level. In particular, two different models were developed, considering, respectively, PA (adherence to the WHO guidelines on leisure-time PA vs. not) and fruits/vegetables consumption (at least three portions of fruits and/or vegetables per day vs. not) as dependent variables. In each model, independent variables were HL level, sex, age, educational level, occupational status, financial status and nationality. 

All confidence intervals (CI) are calculated taking 95% as the confidence level and an alpha level of 0.05 was considered as significant.

Statistical analyses were supported by Stata 15.0 (STATA Corp LLC, 4905 Lakeway Drive, College Station, TX, USA).

## 3. Results

Among the total sample of 7157 interviewees (51.31% females; age: 45.06 ± 14.03) in Tuscany between 2017 and 2018, 66.55% were employed, 69.85% had a high education level and 56.95% reported a poor economic status. Sufficient HL level was observed in 61.17%, 53.42% eat at least three portions of fruit and/or vegetables per day, and 64.5% of participants were physically active during leisure time. Except for sex, all the variables were significantly associated with HL level (Table 1).

According to the multinomial logistic regression, female sex, poor financial status, foreign nationality and low education were associated with a problematic HL level. In contrast, an inadequate HL level was associated with being 50–69 years old, low education level, foreign nationality, poor financial status and with unemployment or inactive status (Table 2).

In the multivariate logistic regression, participants with a low education level, poor financial status and inadequate HL level had a decreased adjusted odds ratio (AdjOR) for eating three or more portions of vegetables and fruit per day. On the contrary, female sex, being between 50 and 60 years old, being inactive and unemployment status significantly increased the probability of eating three or more portions per day of fruits or vegetables (Table 3).

Regarding PA, 35.5% of individuals engaged in less than the recommended amount of leisure time PA (Table 1). In the multivariate analysis sex, age, education, nationality, financial status, occupation and HL level were significantly associated with PA in leisure time. In particular, female interviewees followed WHO PA guidelines less than men. Moreover, people aged 50–60 years old, with low education, a poor financial status, foreign nationality and an inadequate HL level less likely engaged in sufficient PA during their leisure time. In contrast, respondents not in the labour force were more likely to engage in PA compared to those employed (Table 4).

## 4. Discussion

The aims of our study were to describe HL level and its relationship with sociodemographic factors and with certain health-related behaviours in a representative sample of the Tuscan population.

The results show inadequate or problematic HL level in about 40% of the sample. Concerning HL antecedents, a clear gradient in the association with education, nationality and financial status has been observed: the lower the HL level, the stronger the association with low education, poor financial status, and with foreign nationality. Moreover, we found that women were more likely to have a problematic HL level than men, while no significant difference emerged regarding inadequate HL level. On the contrary, subjects aged between 50 and 69 were more likely to have inadequate HL, but no difference was observed with regard to problematic HL.

Regarding health behaviours, we found that HL was significantly associated with both fruits/vegetables consumption and PA: inadequate HL level was a strong predictor of both eating less than three portions of fruits/vegetables per day and of not engaging in sufficient PA during leisure time. The results are in line with the concept of HL described by Sorensen et al. [7] and with the related logical framework. In fact, HL can be defined as “knowledge, motivation and competences to access, understand, appraise, and apply health information in order to make judgments and take decisions in everyday life concerning healthcare, disease prevention and health promotion to maintain or improve quality of life during the life course”. With this perspective, the lower the HL level, the lower the awareness towards healthy lifestyle, as well as the lower the ability to put into practice the recommendations concerning PA and fruits/vegetables consumption. For this point of view, the results of this study can contribute to confirm—by using population-based data—the logical framework proposed by Sorensen et al. [7].

Nevertheless, the results of our study present many differences with respect to other published research in the field.

First of all, if the distribution of HL level in the population is consistent with results from the European Health Literacy surveys [3] and some Italian studies [29,30], we found higher HL level compared to other Italian studies [20,31,32]. Particularly, Lorini et al. [20] using HLS–EU–Q6 found a prevalence of problematic HL level twice as high as that shown in our study. The difference likely may be explained by the diverse composition of our sample with respect to age: in both studies, people of 18–69 years were included; in the study by Lorini et al. [20] (conducted in Florence, Tuscany), the mean age is higher than the one of the sample included in this study. It is well-known, in fact, that the frequency of low HL increases with age [33,34,35,36]. 

The findings related to the association between sociodemographic factors and HL are similar with those already described by previous studies, except for the association between women and problematic HL level. In the literature, findings about an association between sex and HL level in the general population are unclear [37]. Some studies found that women typically have slightly better HL than men [38,39,40,41,42,43]. On the contrary, other studies found no significant sex differences for HL [36,44,45,46]. This may be a problematic issue revealing inequalities in accessing health information and services by Tuscan women; however, these data should be interpreted with caution. In fact, Lee et al. [47] reported that men had a significant tendency to overestimate their comprehension of health information when HL is measured with self-assessment tools.

Regarding healthy lifestyles, we observed some differences in the way sociodemographic factors are related with diet and PA. We observed significant sex differences concerning the examined health behaviours, suggesting that women have a healthier diet than men, while men are more physically active than women; age positively affects diet but affects PA negatively; foreign nationality lowers the likelihood of having sufficient PA, but no differences are observed in relation to diet. On the contrary, high HL levels seem to be positively associated with the adoption of both a healthy diet and a more active lifestyle, strengthening its key role in reducing sociodemographic inequalities.

The role of HL in influencing dietary pattern is still unclear. While some studies found an association between healthy diet and HL levels [48], others did not [49,50]. Furthermore, in past years, Nutrition Literacy (NL) and Food Literacy (FL) were used as synonyms of HL, while in recent years, NL and FL studies have become two new distinct concepts to better identify a set of knowledge, competencies, and abilities that are necessary for people to use information to achieve a healthy diet [51,52]. Consequently, due to the different definitions and assessment tools used for each concept, it is difficult to compare our results with evidence in the literature. For instance, as reported by Vettori et al. [53], the Italian NL assessment tool (NLit–IT) score is not significantly associated with the score of the Italian version of HLS–EU–Q6.

It is worth noting that only inadequate HL level seems to negatively impact PA and diet, while problematic HL level does not seem to have any significant impact compared to a sufficient HL level. Generally, in the literature, problematic and inadequate HL level are addressed as a unicum using the term “low HL”. However, knowing the different effect of problematic and inadequate HL level may have important consequences in the interpretation of research results. Moreover, this will impact the analysis of the efficacy of interventions aimed at improving the HL of the population in order to promote PA and healthy diet.

Our study includes a large representative sample of 7157 subjects. The use of a multinomial logistic regression analysis allowed us to distinguish the different relationships that may be present between HL levels and sociodemographic factors, particularly regarding sex and age. Moreover, thanks to the adoption of multivariate logistic regression models, we have highlighted possible different effects that inadequate and problematic levels of HL may have on health behaviours. 

However, our work presents some limitations. First, PASSI surveillance did not take into account the population over 69 years old; this aspect limited the generalisability of the results to the elderly. Furthermore, the study presented the usual limits of cross-sectional design in testing associations: exposure is determined simultaneously with the outcome and it is not possible to determine the chronological order of events. Longitudinal studies are needed to better understand the relationship between sociodemographic factors, HL, and healthy behaviours. Lastly, there is no ‘gold standard’ for measuring PA and diet, thus limiting the comparability of our findings with other studies. Moreover, self-reported PA and fruits/vegetables consumption can be subjected to recall bias and social approval bias [54,55]. In particular, social desirability bias could occur because of the reticence to declare a behaviour that is prone to social disapproval or to report a major compliance to desirable behaviours, leading to possible overestimation of fruit and vegetables consumption or physical activity. However, after accounting for these limitations, research on validity and reliability supported the utility of the data obtained by BRFS such as PASSI [56].

## 5. Conclusions

In conclusion, our findings showed that an inadequate level of HL could negatively affect PA and diet, independent of the other sociodemographic conditions, confirming the role of HL as a relevant social determinant of health. Furthermore, our findings clearly showed that people who are in socioeconomically disadvantaged groups present a lower level of HL. According to Gazmararian et al. [57], monitoring the incidence of social determinants—including HL—among the population of a given country is a mainstay for planning patient-centred healthcare services, policies, and achieving a more public health-literate society. In this sense, the inclusion of a measurement tool of HL into behavioural or risk factor surveillance systems seems to be meaningful for developing broader and more effective interventions of prevention and health promotion.

## Figures and Tables

**Table 1:** Descriptive analysis of collected variables (total sample, subjects with sufficient, problematic and inadequate health literacy level).

Variables	Total % (N = 7157) *	Health Literacy Level % ^§^			
		Sufficient	Problematic	Inadequate	*p* °
		61.17	30.22	8.62	
Age					<0.001
18–34	24.7	63.82	29.22	6.96	
35–49	33.73	63.13	29.14	7.72	
50–69	41.57	57.99	31.68	10.33	
Sex					0.055
Male	48.69	62.27	28.81	8.92	
Female	51.31	60.12	31.55	8.33	
Occupation status					<0.001
Employed	66.55	63.73	29.29	6.97	
Unemployed	7.361	53.82	32.53	13.66	
Inactive	26.09	56.65	31.94	11.41	
Education level					<0.001
Low	30.15	49.53	35.39	15.08	
High	69.85	66.18	28.01	5.82	
Financial Status					<0.001
Poor	43.05	52.93	33.81	13.26	
Good	56.95	67.94	26.77	5.29	
Nationality					<0.001
Italian	93.39	62.54	30.02	7.44	
Foreign	6.61	40.81	33.66	25.52	
Physical Activity					<0.001
Active	64.5	62.69	30.80	6.50	
Non-Active	35.5	58.42	29.19	12.40	
Fruits and Vegetables consumption					0.001
<3 portions	46.58	59.34	30.75	9.91	
≥ 3 portions	53.42	62.74	29.77	7.49	

* column percentage; ^§^ row percentage; ° chi-squared test.

**Table 2:** Multinomial logistic regression analysis: association of health literacy level with sociodemographic factors; RRR: Relative Risk Ratio; CI: Confidence Interval; HL: Health Literacy.

	Sufficient HL	Problematic HL			Inadequate HL		
Sociodemographic Factors	Reference Category	RRR	*p*	95% CI	RRR	*p*	95% CI
Age							
18–34 *	–	–	–	–	–	–	–
35–49	1.00	1.01	0.909	0.86–1.18	1.25	0.102	0.96–1.64
50–69	1.00	1.09	0.286	0.93–1.26	1.57	0.000	1.22–2.03
Sex							
Male *	–	–	–	–	–	–	–
Female	1.00	1.13	0.037	1.01–1.27	0.86	0.128	0.71–1.04
Education level							
High *	–	–	––	–	–	–	–
Low	1.00	1.50	0.000	1.31–1.71	2.34	0.000	1.92–2.87
Occupation status							
Employed *	–	–	–	–	–	–	–
Unemployed	1.00	1.16	0.207	0.92–1.45	1.66	0.002	1.20–2.30
Inactive	1.00	1.08	0.290	0.94–1.24	1.59	0.000	1.26–1.99
Financial Status							
Good *	–	–	–	–	–	–	–
Poor	1.00	1.47	0.000	1.30–1.65	2.43	0.000	1.98–2.97
Nationality							
Italian *	–	–	–	–	–	–	–
Foreign	1.00	1.49	0.001	1.17–1.91	4.36	0.000	3.29–5.77

* Reference Group.

**Table 3:** Multivariate logistic regression analysis: predictors of eating at least 3 portions of fruits/vegetables per day (the logistic regression model includes all the covariates in the table); AdjOR: Adjusted Odds Ratio; CI: Confidence Interval; HL: Health Literacy.

Eating at Least 3 Portions Fruits/Vegetables per Day	Prevalence %	*AdjOR*	*p*	95% CI
Total	53.42	–	–	–
Age				
18–34 *	49.52	–	–	–
35–49	51.47	1.16	0.035	1.01–1.33
50–69	59.33	1.58	0.000	1.38–1.80
Sex				
Male *	47.43	–	–	–
Female	60.92	1.69	0.000	1.52–1.87
Education level				
High *	55.4	–	–	–
Low	51.71	0.87	0.023	0.77–0.98
Occupation status				
Employed *	52.38	–	–	–
Unemployed	54.13	1.27	0.024	1.03–1.56
Inactive	58.94	1.21	0.004	1.06–1.37
Financial Status				
Good *	58.15	–	–	–
Poor	49.96	0.74	0.000	0.67–0.83
Nationality				
Italian *	54.52	–	–	–
Foreign	50.17	1.02	0.881	0.82–1.26
HL level				
Sufficient *	54.81	–	–	–
Problematic	52.62	0.94	0.301	0.84–1.06
Inadequate	46.43	0.75	0.003	0.62–0.91

* Reference Group.

**Table 4:** Multivariate logistic regression analysis: predictors of eating at least 3 portions of fruits/vegetables per day (the logistic regression model includes all the covariates in the table); AdjOR: Adjusted Odds Ratio; CI: Confidence Interval; HL: Health Literacy.

Physically Active	Prevalence %	AdjOR	*p*	95% CI
Total	64.5	–	–	–
Age				
18–34 *	68.5	–	–	–
35–49	62.49	0.88	0.077	0.76–1.01
50–69	62.3	0.86	0.031	0.74–0.99
Sex				
Male *	64.15	–	–	–
Female	63.63	0.88	0.028	0.79–0.99
Education level				
High *	67.73	–	–	–
Low	55.8	0.66	0.000	0.59–0.75
Occupation status				
Employed *	62.82	–	–	–
Unemployed	62.45	1.17	0.152	0.94–1.46
Inactive	67.21	1.40	0.000	1.23–1.61
Financial Status				
Good *	67.75	–	–	–
Poor	59.46	0.78	0.000	0.70–0.87
Nationality				
Italian *	65.05	–	–	–
Foreign	49.12	0.66	0.000	0.53–0.82
HL level				
Sufficient *	66.11	–	–	–
Problematic	65.73	1.09	0.150	0.97–1.24
Inadequate	48.81	0.60	0.000	0.50–0.73

* Reference Group.

## Data Availability

The data presented in this study are available on request from the corresponding author. The data are not publicly available due to privacy restrictions.
